# Superoxide Mediates Depressive Effects Induced by Hydrogen Sulfide in Rostral Ventrolateral Medulla of Spontaneously Hypertensive Rats

**DOI:** 10.1155/2015/927686

**Published:** 2015-05-11

**Authors:** Haiyun Yu, Haiyan Xu, Xiaoni Liu, Nana Zhang, Anqi He, Jerry Yu, Ning Lu

**Affiliations:** ^1^Department of Physiology and Pathophysiology, Shanghai Medical College, Fudan University, Yixueyuan Road 138, Xuhui District, Shanghai 200032, China; ^2^Beijing Electric Power Hospital, Capital Medical University, China; ^3^Department of Medicine, University of Louisville, KY, USA

## Abstract

Hydrogen sulfide (H_2_S) plays a crucial role in the regulation of blood pressure and oxidative stress. In the present study, we tested the hypothesis that H_2_S exerts its cardiovascular effects by reducing oxidative stress via inhibition of NADPH oxidase activity in the rostral ventrolateral medulla (RVLM). We examined cell distributions of cystathionine-*β*-synthase (CBS) and effects of H_2_S on reactive oxygen species (ROS) and mean arterial blood pressure (MAP) in spontaneously hypertensive rats (SHRs). We found that CBS was expressed in neurons of the RVLM, and the expression was lower in SHRs than in Wistar-Kyoto rats. Microinjection of NaHS (H_2_S donor), S-adenosyl-l-methionine (SAM, a CBS agonist), or Apocynin (NADPH oxidase inhibitor) into the RVLM reduced the ROS level, NADPH oxidase activity, and MAP, whereas microinjection of hydroxylamine hydrochloride (HA, a CBS inhibitor) increased MAP. Furthermore, intracerebroventricular infusion of NaHS inhibited phosphorylation of p47^phox^, a key step of NADPH oxidase activation. Since decreasing ROS level in the RVLM reduces MAP and heart rate and increasing H_2_S reduces ROS production, we conclude that H_2_S exerts an antihypertensive effect via suppressing ROS production. H_2_S, as an antioxidant, may be a potential target for cardiovascular diseases.

## 1. Introduction

H_2_S is an important gasotransmitter as are nitric oxide, carbon monoxide, and ammonium [1–4]. Endogenous H_2_S is produced by three enzymes, cystathionine-*β*-synthase (CBS), cystathionine-*γ*-lyase (CSE), and 3-mercaptopyruvate sulfurtransferase in conjunction with cysteine aminotransferase. In the brain, the production of H_2_S is mainly catalyzed by CBS [5–7].

H_2_S participates in the regulation of numerous physiological functions [8]. In the central nervous system (CNS), H_2_S exerts important multifaceted neuromodulatory effects. Evidence highlights a crucial role of H_2_S in the development of hypertension. For example, Yang et al. found that genetic deletion of CSE in mice resulted in hypertension [9]. Systemic administration of H_2_S donors and precursors decreased mean arterial pressure (MAP) in various models of hypertension [10–13]. Nevertheless, mediation of H_2_S in the cardiovascular center has been controversial [14–16].

The rostral ventrolateral medulla (RVLM), where sympathetic premotor neurons are located, is connected with other cardiovascular nuclei that regulate sympathetic nerve activity [17, 18]. Reactive oxygen species (ROS) in the RVLM plays a pivotal role in the pathogenesis of hypertension and heart failure [19–22]. Overproduction of O_2_^∙−^ and H_2_O_2_ contributes to hypertension by increasing sympathetic outflow to blood vessels [23–25]. Thus, upregulation of endogenous antioxidants is potentially an effective therapeutic strategy for cardiovascular diseases. A recent study indicates that neurons were protected by the antioxidant effect of H_2_S [26]. However, its role in central cardiovascular mechanisms remains unclear. The present study was undertaken to assess the hypothesis that H_2_S exerts antihypertensive effects by decreasing ROS production by inhibiting NADPH oxidase activity in the RVLM.

## 2. Materials and Methods

### 2.1. Animals and Agents

Male spontaneously hypertensive rats (SHRs), weighing 280–310 g, were supplied by the Experimental Animal Center of Department of Physiology and Pathophysiology, Shanghai Medical College, Fudan University. They were housed socially (3–5 per cage with food and water ad libitum) and kept on a 12-hour light/12-hour dark cycle. Studies were approved by the Ethics Committee of Experimental Research, Shanghai Medical College, Fudan University. NaHS, lucigenin, S-adenosyl-l-methionine (SAM), and hydroxylamine hydrochloride (HA) were purchased from Sigma. Apocynin (APO) was purchased from Calbiochem, and the antibodies (anti-CBS, anti-MAP-2, anti-GFAP, and p47^phox^ antibodies) were purchased from Jackson and Abcom. BCA kits were purchased from Beyotime.

### 2.2. Immunofluorescence Staining and Laser Confocal Microscopy

Rats were anaesthetized with chloral hydrate (300 mg/kg ip) and then transcardially perfused with 150 mL saline followed by 250 mL 4% paraformaldehyde in 0.1 M sodium phosphate buffer (0.1 M PB; pH 7.4). Brains were rapidly dissected and postfixed in the same fixative solution at 4°C for 6 h and then transferred sequentially into 20 and 30% sucrose in 0.1 M PB for cryoprotection. Transverse serial medullary sections (30 *μ*m thick) were cut with a microtome (Reichert-Jung) 1.5–1.7 mm rostral to the obex according to Paxinos and Watson's atlas. Sections were immersed in 4% paraformaldehyde for 10 min followed by 6 × 5 min washing in 0.01 M phosphate-buffered saline (0.01 M PBS; pH 7.4). Free floating sections were incubated in 2% BSA and 0.2% Triton X-100 in 0.01 M PBS for 30 min at 37°C to eliminate nonspecific staining, and they were then exposed to antibodies for 1 h at 37°C, plus an additional 24 h at 4°C for the first primary antibody. The sections were then washed three times in TBST and incubated with fluorescent secondary antibodies for 60 min for confocal microscopy (Zeiss LSM510, Jena, Germany).

### 2.3. Microinjection into the RVLM

Rats were anesthetized with a mixture of urethane (700 mg/kg) and *α*-chloralose (35 mg/kg) and intubated to facilitate ventilation. The left femoral artery was cannulated to monitor blood pressure and heart rate. Body temperature was maintained between 37°C and 37.5°C during the experiment with a temperature-controlled table. Then, rats were mounted in a stereotaxic frame and a micropipette tip (outer diameter 10–30 *μ*m) was inserted into the RVLM [27] for microinjection (1.8 to 2.1 mm lateral to the midline, 2.6 to 3.3 mm caudal to interaural line, and 0.3 to 0.9 mm from the ventral surface). Injection sites were confirmed histologically.

### 2.4. Intracerebroventricular Infusion

A lateral ventricular cannula was implanted after the general surgical procedures. Anesthetized rats were placed in a stereotaxic frame and a small hole was made in the skull (1.2–1.4 mm lateral to midline and 0.8–1.0 mm posterior to bregma). A 10 mm stainless steel guide cannula (22 gauges) was lowered 4 mm below the surface of the skull and fixed with cranioplastic cement. A stainless steel injector was introduced through the guide cannula to 0.5 mm beyond its tip.

### 2.5. Measurement of Superoxide Production

The lucigenin-enhanced chemiluminescence assay was used to determine superoxide production as previously described [28]. After infusion of various agents, the ventrolateral medulla was removed and homogenized in a 0.02 mol/L phosphate-buffered saline (PBS), pH 7.4, containing 0.01 mM EDTA. The homogenate was centrifuged at 1000 g for 10 min at 4°C to remove nuclei and cell debris. Supernatant was obtained immediately for O_2_^∙−^ measurement. Background chemiluminescence was used for assessing O_2_^∙−^. An aliquot of supernatant (100 *μ*L) was then added to buffer (2 mL) containing lucigenin (5 *μ*mol/L) and measured for chemiluminescence. O_2_^∙−^ production was calculated and expressed as mean light unit per mg protein.

### 2.6. Measurement of NADPH Oxidase Activity

NADPH oxidase activity in the ventrolateral medulla was determined by a luminescence assay. The preparation was identical to that for O_2_^∙−^. The luminescent assay was performed in PBS buffer containing 0.01 M/L EGTA and 5 *μ*M/L lucigenin as the electron acceptor and 100 mM/L NADPH as the substrate. After dark adaptation, background counts were recorded and a tissue homogenate (1 *μ*L protein sample) was added. The chemiluminescence value was recorded at 1 min intervals for 30 min. O_2_^∙−^ production was measured after addition of NADPH to the incubation medium with and without a flavoprotein inhibitor of NADPH oxidase, Apocynin.

### 2.7. Western Blot Analysis

After having been anesthetized, rat medullas were rapidly removed and frozen immediately in liquid nitrogen until being homogenized in cell lysis buffer, followed by centrifugation at 12000 g for 15 min at 4°C. The supernatant was obtained for protein concentration. Then, protein samples were separated by 10% SDS-PAGE and transferred onto a nitrocellulose membrane. After blocking at room temperature in 5% BSA for 1 h, the membrane was incubated with various primary antibodies at 4°C overnight and then washed three times in TBST buffer and incubated with 1 : 5000 dilutions of anti-mouse IgG. Visualization was made with an enhanced chemiluminescent kit. Band densities on Western blot were quantified with *β*-actin as internal control.

### 2.8. Statistical Analysis

Data were analyzed with statistical software SigmaSTat (SPSS 17.0) and expressed as the mean ± SEM. One-way ANOVA with repeated measures was used as appropriate to assess group means followed by the Bonferroni post hoc tests. Probability values of *P* < 0.05 were considered significant.

## 3. Results

We studied H_2_S induced antihypertensive effects in SHRs by examining molecular mechanisms involved in the RVLM from 4 different aspects.

### 3.1. Expression of CBS

**Figure 1:**
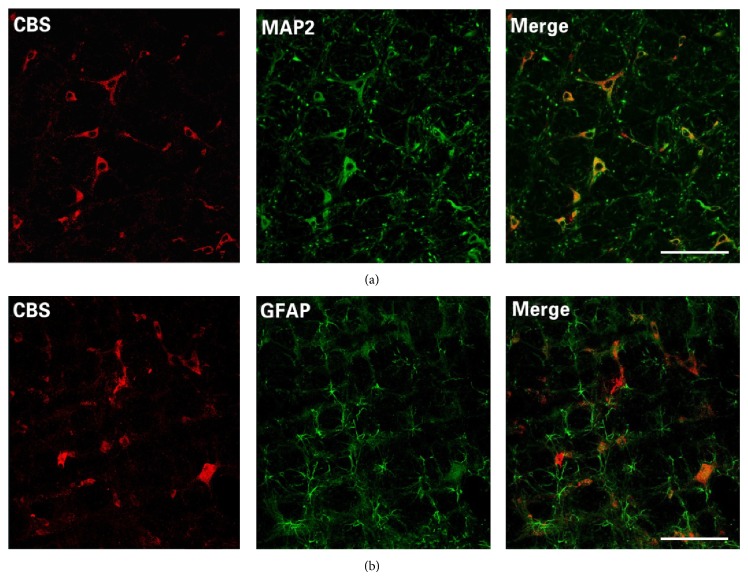
CBS expression in RVLM neurons. Confocal images showed that CBS immunoreactivity is colocalized with a neuronal marker (MAP2: upper panels) but not a glia marker (GFAP: lower panels).

**Figure 2:**
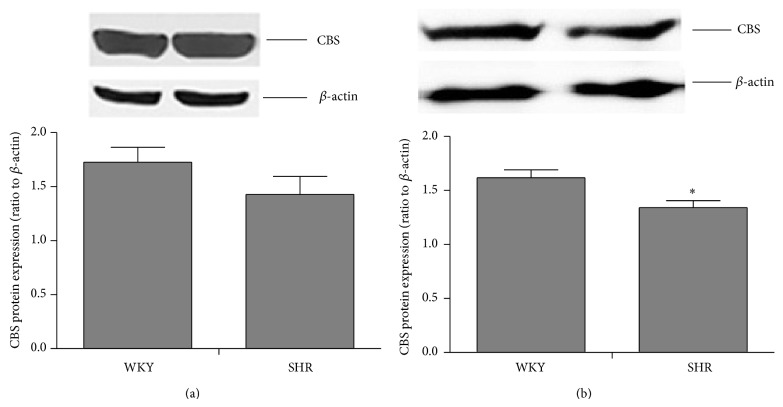
CBS is expressed less in the RVLM of hypertensive rats. CBS protein expression in WKY rats (*n* = 5) and SHR (*n* = 6) at 8 weeks (a) and 17 weeks (b). ^*^ *P* < 0.05, SHR versus WKY. Please note that the difference in CBS expression occurred only at 17 weeks of age, when hypertension developed.

Cellular distribution of CBS was identified by immunofluorescent stain coupled with laser confocal microscopy. CBS immunoreactivity was found in neuronal cells, but not in glia cells (Figure 1). CBS expressions in the RVLM were confirmed by Western blot assay, which were the same in SHRs and WKY rats at 8 weeks of age; however, expression was lower in SHRs at 17 weeks of age (Figure 2).

### 3.2. Effects of H_2_S on MAP and HR

**Figure 3:**
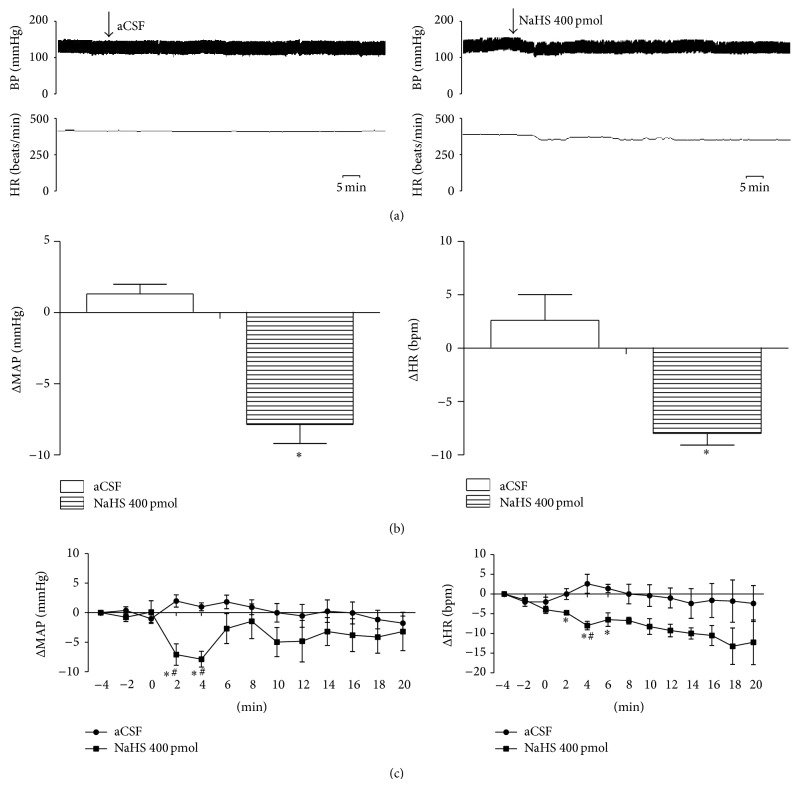
Microinjection of NaHS (400 pmol) into the RVLM decreased MAP and HR in SHRs. (a) Typical MAP and HR traces in response to the microinjection. (b) Maximal changes detected during the response. (c) Time courses of MAP and HR in response to microinjections of aCSF, artificial cerebral spinal fluid (*n* = 5), or NaHS (*n* = 4). ^*^ *P* < 0.05 versus aCSF control group.

**Figure 4:**
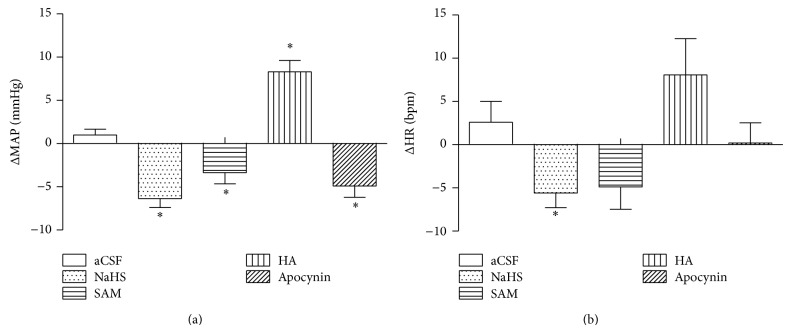
Maximal responses in MAP (a) and HR (b) to microinjections of different agents into the RVLM in SHRs. aCSF (control), *n* = 5; NaHS (H_2_S donor), *n* = 7; SAM (a CBS agonist), *n* = 5; HA (a CBS inhibitor), *n* = 5; and APO (NADPH oxidase inhibitor), *n* = 5. ^*^ *P* < 0.05 versus aCSF group.

Microinjection of NaHS (400 pmol/0.1 *μ*L) into the RVLM significantly decreased mean arterial blood pressure (MAP) and heart rate (HR) (Figure 3). Typically, MAP returned to baseline within 10–20 min. Similarly, microinjection of S-adenosyl-l-methionine (SAM, a CBS agonist, 10 pmol/0.1 *μ*L) or Apocynin (APO, a NADPH oxidase inhibitor, 10 nmo/0.1 *μ*L) decreased MAP. On the other hand, microinjection of hydroxylamine hydrochloride (HA, a CBS inhibitor, 9 nmol/0.1 *μ*L) increased MAP (Figure 4). These results support a link between H_2_S and ROS and provide novel evidence for regulation of hemodynamics by exogenous and endogenous H_2_S in the RVLM.

### 3.3. Effect of H_2_S on O_ 2 _^∙−^ Production and NADPH Oxidase Activity

**Figure 5:**
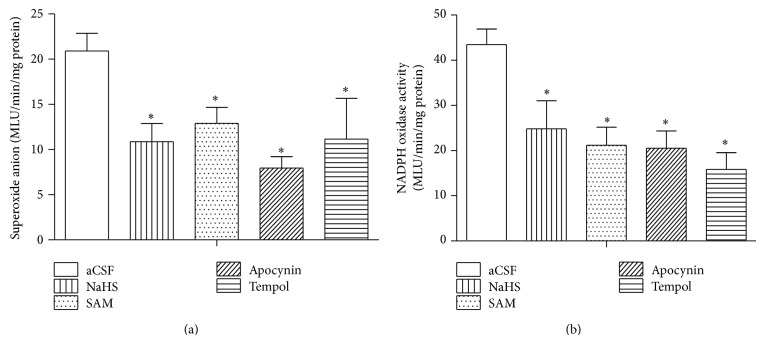
Infusion of various depressive agents suppressed NADPH oxidase activity and superoxide production in the RVLM of SHRs. Tissue levels of superoxide anion (a) and NADPH oxidase activity (b) after infusion of aCSF (artificial cerebral spinal fluid, *n* = 9), NaHS (H_2_S donor, *n* = 5), Apocynin (NADPH oxidase inhibitor, *n* = 5), SAM (a CBS agonist, *n* = 4), or Tempol (SOD mimetic, *n* = 4). ^*^ *P* < 0.05 versus aCSF group.

Microinjection of NaHS (400 pmol), SAM (10 pmol/0.1 *μ*L), APO (10 nmol/0.1 *μ*L), or Tempol (a SOD mimetic, 50 nmol/0.1 *μ*L) decreased the level of superoxide anion (O_2_^∙−^) in the RVLM (Figure 5(a)). NADPH oxidase is a major enzyme for superoxide production in the brain. To determine whether the decrease of ROS results from inhibition of this enzyme, we assessed the activity of NADPH oxidase and found that microinjection of NaHS, SAM, and APO decreased NADPH oxidase activity significantly (Figure 5(b)).

### 3.4. Effect of H_2_S on Phosphorylation of NADPH Oxidase

**Figure 6:**
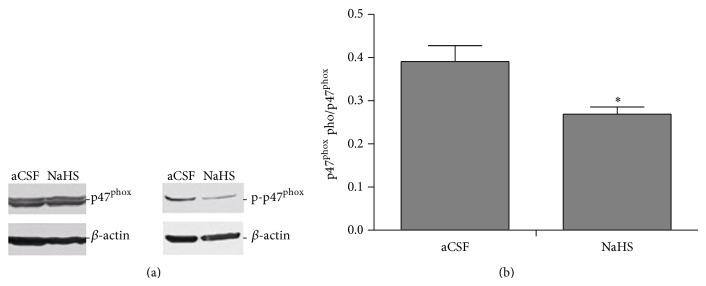
Exogenous H_2_S suppressed p47^phox^ phosphorylation of NADPH oxidase in the RVLM. Western blots show that p47^phox^ phosphorylated/p-47 protein levels after intracerebroventricular infusion of aCSF or NaHS. Representative gel: (a) representative densitometric analysis and (b) group data (*n* = 5); ^*^ *P* < 0.05 versus aCSF group.

Phosphorylation of p47^phox^ subunit is an important step for activation of NADPH oxidase. Thus, we examined the effect of intracerebroventricular infusion of NaHS on phosphorylation of p47^phox^ serine residues. We found that NaHS significantly decreased serine phosphorylation of p47^phox^ in the RVLM (Figure 6), supporting that NaHS reduces production of superoxide via suppression of serine phosphorylation of p47^phox^.

## 4. Discussion

Our results provide the first evidence demonstrating that NADPH oxidase derived superoxide mediates the antihypertensive effects of H_2_S in the RVLM. Our statement is supported by the following 4 findings: (1) CBS was expressed in RVLM neurons, which provides an anatomical basis for the regulation; (2) increasing exogenous or endogenous H_2_S in the RVLM decreased NADPH oxidase activity, superoxide anion, and MAP; (3) decreasing ROS produced the same depressive effects; (4) infusion of NaHS inhibited phosphorylation of p47^phox^, a key step of NADPH oxidase activation.

H_2_S can be produced endogenously in various parts of the body in the heart, kidney, liver, and CNS. CBS is significantly expressed in the CNS, especially in the hippocampus and cerebellum, as well as the cerebral cortex and brain stem [29]. CBS has been identified in astrocytes, microglia, and neurons [30–32]. However, its cellular distribution in the RVLM is unknown. Our data revealed that CBS proteins were expressed mainly in RVLM neurons, but not glial cells (Figure 1). Furthermore, the level of CBS proteins in the RVLM was lower in SHRs than in WKY rats (Figure 2), which is consistent with a recent report of intracerebroventricular infusion with NaHS [33]. It is interesting to note that the difference in CBS expression did not occur until hypertension developed.

Accumulating evidence highlights the crucial role of H_2_S homeostasis in hypertension. A transient hypotensive effect was first reported in anesthetized rats with administration of H_2_S donors [4]. The CSE-L-cysteine pathway was downregulated and H_2_S was effective in reducing MAP and vascular remodeling in SHRs [12]. However, direct evidence for blood pressure control was reported in CES gene deficient mice [9]. Administration of H_2_S donors and precursors decreases MAP in various hypertensive models (chronic inhibition of nitric oxide synthase, two-kidney-one-clip, and SHRs) [9–13]. The antihypertensive effect of H_2_S has also been studied by infusion of NaHS into the RVLM cardiovascular center [15, 31]. The RVLM receives neuronal input from the paraventricular nucleus, solitary tracts nuclei, and so forth and then sends the signal to the spinal cord to regulate MAP and HR [17, 18]. Microinjection of NaHS (200, 400, and 800 pmol) into the RVLM decreases MAP, HR, and renal sympathetic nerve activity in a dose-dependent manner in SD rats [31]. Consistent with this study, our current results show that microinjection of NaHS (400 pmol) into the RVLM significantly decreased MAP and HR. Furthermore, we demonstrated that increased endogenous H_2_S by microinjection of SAM (a CBS agonist) or decreased ROS by infusion of Apocynin produced the same depressive effects, while microinjection of HA (a CBS inhibitor) increased MAP, supporting that H_2_S is a negative regulator for blood pressure in the RVLM.

Overproduction of ROS is critical for the pathogenesis of cardiovascular diseases, including hypertension and heart failure [21, 34, 35]. The baseline ROS, including O_2_^∙−^ and H_2_O_2_, in the RVLM is elevated in hypertensive animals [36, 37]. Elevated ROS in the brain increased MAP and sympathoexcitation, probably because of an upregulation of AT_1_ receptor and NADPH oxidase [38, 39]. It has been reported that NO exerts antihypertensive effects by inhibiting NADPH oxidase and thus reduces O_2_^∙−^ production [40–42]. Since H_2_S also exerts an antihypertensive effect, we speculate that H_2_S operates with the same mechanism. Indeed, exogenous (microinjection of NaHS) and endogenous (microinjection of SAM) H_2_S decreased NADPH oxidase activity and O_2_^∙−^ production. Our hypothesis is further supported by the decreased O_2_^∙−^ with the addition of Apocynin (a NADPH oxidase inhibitor) or Tempol (a cell membrane-permeable SOD mimetic). It is worth noting that increasing H_2_S by microinjection of NaHS or SAM decreased MAP and HR, while decreasing ROS by microinjection of Apocynin decreased MAP only. We speculate that H_2_S may exert additional influence on HR through another mechanism. Further studies are needed to verify this plausibility.

ROS can be produced by xanthine oxidase, cytochrome P450, mitochondrial respiratory chain enzyme, or NADPH oxidase, which is the major enzyme for superoxide production in the brain. Its activation is initiated by serine phosphorylation of its cytosolic regulatory p47^phox^ subunit [43, 44]. We found that NaHS infusion significantly decreased phosphorylated p47^phox^ levels in the RVLM, which would decrease enzyme activity of NADPH oxidase and superoxide production. Furthermore, microinjection of Apocynin decreased blood pressure. Muzaffar et al. observed that H_2_S downregulated NADPH oxidase and inhibited O_2_^∙−^ formation in pulmonary arterial endothelial cells, and this effect could be canceled by inhibitors of PKA, but not by inhibitors of PKG, indicating that the effect of H_2_S on NADPH oxidase may be mediated by the adenylyl cyclase-cAMP-PKA pathway [45]. Taken together, our results suggest that NADPH oxidase-derived superoxide mediates H_2_S induced central depressive effects. Since NADPH oxidase is composed of membrane-bound (gp91^phox^ and p22^phox^) and cytoplasmic (p47^phox^, p40^phox^, and p67^phox^) subunits and small molecules (GTPase Rac1 and/or Rac2), the role of each component of the enzyme in the mediation requires further exploration.

In summary, present studies demonstrated that the H_2_S metabolic system was present in the RVLM, and central administration of H_2_S into the RVLM decreased phosphorylation of NADPH oxidase, NADPH oxidase activity, and O_2_^∙−^ production and reduced MAP and HR in SHRs, whereas decreasing H_2_S by microinjection of a CBS antagonist increased MAP. Yet our data support that H_2_S in the RVLM may decrease MAP mediated through NADPH oxidase, which is largely based on correlation, and a direct mediation is not conclusive. Further studies are still needed. Nevertheless, since overproduction of superoxide in the CNS is involved in the etiology of hypertension, we expect that the H_2_S-NADPH oxidase-superoxide system may be an effective therapeutic target in preventing hypertension.

## References

[1] Wang R. (2014). Gasotransmitters: growing pains and joys. *Trends in Biochemical Sciences*.

[2] Tan B. H., Wong P. T.-H., Bian J.-S. (2010). Hydrogen sulfide: a novel signaling molecule in the central nervous system. *Neurochemistry International*.

[3] Wang R. (2002). Two's company, three's a crowd: can H_2_S be the third endogenous gaseous transmitter?. *The FASEB Journal*.

[4] Zhao W., Zhang J., Lu Y., Wang R. (2001). The vasorelaxant effect of H_2_S as a novel endogenous gaseous K_ATP_ channel opener. *The EMBO Journal*.

[5] Wang R. (2010). Hydrogen sulfide: the third gasotransmitter in biology and medicine. *Antioxidants and Redox Signaling*.

[6] Kimura H., Nagai Y., Umemura K., Kimura Y. (2005). Physiological roles of hydrogen sulfide: synaptic modulation, neuroprotection, and smooth muscle relaxation. *Antioxidants and Redox Signaling*.

[7] Moore P. K., Bhatia M., Moochhala S. (2003). Hydrogen sulfide: from the smell of the past to the mediator of the future?. *Trends in Pharmacological Sciences*.

[8] Wang R. (2012). Physiological implications of hydrogen sulfide: a whiff exploration that blossomed. *Physiological Reviews*.

[9] Yang G., Wu L., Jiang B. (2008). H_2_S as a physiologic vasorelaxant: hypertension in mice with deletion of cystathionine *γ*-lyase. *Science*.

[10] Stubbert D., Prysyazhna O., Rudyk O., Scotcher J., Burgoyne J. R., Eaton P. (2014). Protein kinase g I*α* oxidation paradoxically underlies blood pressure lowering by the reductant hydrogen sulfide. *Hypertension*.

[11] Lu M., Liu Y.-H., Goh H. S. (2010). Hydrogen sulfide inhibits plasma renin activity. *Journal of the American Society of Nephrology*.

[12] Yan H., Du J., Tang C. (2004). The possible role of hydrogen sulfide on the pathogenesis of spontaneous hypertension in rats. *Biochemical and Biophysical Research Communications*.

[13] Zhong G., Chen F., Cheng Y., Tang C., Du J. (2003). The role of hydrogen sulfide generation in the pathogenesis of hypertension in rats induced by inhibition of nitric oxide synthase. *Journal of Hypertension*.

[14] Liu W. Q., Chai C., Li X. Y. (2011). The cardiovascular effects of central hydrogen sulfide are related to K(ATP) channels activation. *Physiological Research*.

[15] Streeter E., Al-Magableh M., Hart J. L., Badoer E. (2011). Hydrogen sulfide in the RVLM and PVN has no effect on cardiovascular regulation. *Frontiers in Physiology*.

[16] Dawe G. S., Han S. P., Bian J.-S., Moore P. K. (2008). Hydrogen sulphide in the hypothalamus causes an ATP-sensitive K^+^ channel-dependent decrease in blood pressure in freely moving rats. *Neuroscience*.

[17] Guyenet P. G. (2006). The sympathetic control of blood pressure. *Nature Reviews Neuroscience*.

[18] Sved A. F., Ito S., Sved J. C. (2003). Brainstem mechanisms of hypertension: role of the rostral ventrolateral medulla. *Current Hypertension Reports*.

[19] Peterson J. R., Sharma R. V., Davisson R. L. (2006). Reactive oxygen species in the neuropathogenesis of hypertension. *Current Hypertension Reports*.

[20] Zimmerman M. C., Davisson R. L. (2004). Redox signaling in central neural regulation of cardiovascular function. *Progress in Biophysics and Molecular Biology*.

[21] Gao L., Wang W., Li Y.-L. (2004). Superoxide mediates sympathoexcitation in heart failure: roles of angiotensin II and NAD(P)H oxidase. *Circulation Research*.

[22] Ross C. A., Ruggiero D. A., Park D. H. (1984). Tonic vasomotor control by the rostral ventrolateral medulla: effects of electrical or chemical stimulation of the area containing C1 adrenaline neurons on arterial pressure, heart rate, and plasma catecholamines and vasopressin. *The Journal of Neuroscience*.

[23] Zacchigna S., Lambrechts D., Carmeliet P. (2008). Neurovascular signalling defects in neurodegeneration. *Nature Reviews Neuroscience*.

[24] Macias M., Dwornik A., Ziemlinska E. (2007). Locomotor exercise alters expression of pro-brain-derived neurotrophic factor, brain-derived neurotrophic factor and its receptor TrkB in the spinal cord of adult rats. *European Journal of Neuroscience*.

[25] Schinder A. F., Poo M.-M. (2000). The neurotrophin hypothesis for synaptic plasticity. *Trends in Neurosciences*.

[26] Samhan-Arias A. K., Garcia-Bereguiain M. A., Gutierrez-Merino C. (2009). Hydrogen sulfide is a reversible inhibitor of the NADH oxidase activity of synaptic plasma membranes. *Biochemical and Biophysical Research Communications*.

[27] Paxinos G., Watson C. (1998). *The Rat Brain in Stereotaxic Coordinates*.

[28] Tarpey M. M., Wink D. A., Grisham M. B. (2004). Methods for detection of reactive metabolites of oxygen and nitrogen: in vitro and in vivo considerations. *The American Journal of Physiology—Regulatory Integrative and Comparative Physiology*.

[29] Abe K., Kimura H. (1996). The possible role of hydrogen sulfide as an endogenous neuromodulator. *Journal of Neuroscience*.

[30] Leffler C. W., Parfenova H., Basuroy S., Jaggar J. H., Umstot E. S., Fedinec A. L. (2011). Hydrogen sulfide and cerebral microvascular tone in newborn pigs. *The American Journal of Physiology—Heart and Circulatory Physiology*.

[31] Guo Q., Jin S., Wang X.-L. (2011). Hydrogen sulfide in the rostral ventrolateral medulla inhibits sympathetic vasomotor tone through ATP-sensitive K^+^ channels. *Journal of Pharmacology and Experimental Therapeutics*.

[32] Hu L.-F., Wong P. T.-H., Moore P. K., Bian J.-S. (2007). Hydrogen sulfide attenuates lipopolysaccharide-induced inflammation by inhibition of p38 mitogen-activated protein kinase in microglia. *Journal of Neurochemistry*.

[33] Sikora M., Drapala A., Ufnal M. (2014). Exogenous hydrogen sulfide causes different hemodynamic effects in normotensive and hypertensive rats via neurogenic mechanisms. *Pharmacological Reports*.

[34] Campos R. R. (2009). Oxidative stress in the brain and arterial hypertension. *Hypertension Research*.

[35] Hirooka Y. (2008). Role of reactive oxygen species in brainstem in neural mechanisms of hypertension. *Autonomic Neuroscience*.

[36] Chan S. H. H., Tai M.-H., Li C.-Y., Chan J. Y. H. (2006). Reduction in molecular synthesis or enzyme activity of superoxide dismutases and catalase contributes to oxidative stress and neurogenic hypertension in spontaneously hypertensive rats. *Free Radical Biology and Medicine*.

[37] Arsenijevic D., Onuma H., Pecqueur C. (2000). Disruption of the uncoupling protein-2 gene in mice reveals a role in immunity and reactive oxygen species production. *Nature Genetics*.

[38] Koga Y., Hirooka Y., Araki S., Nozoe M., Kishi T., Sunagawa K. (2008). High salt intake enhances blood pressure increase during development of hypertension via oxidative stress in rostral ventrolateral medulla of spontaneously hypertensive rats. *Hypertension Research*.

[39] Fujita M., Ando K., Nagae A., Fujita T. (2007). Sympathoexcitation by oxidative stress in the brain mediates arterial pressure elevation in salt-sensitive hypertension. *Hypertension*.

[40] Muzaffar S., Shukla N., Jeremy J. Y. (2005). Nicotinamide adenine dinucleotide phosphate oxidase: a promiscuous therapeutic target for cardiovascular drugs?. *Trends in Cardiovascular Medicine*.

[41] Koupparis A. J., Jeremy J. Y., Muzaffar S., Persad R., Shukla N. (2005). Sildenafil inhibits the formation of superoxide and the expression of gp47^phox^ NAD[P]H oxidase induced by the thromboxane A2 mimetic, U46619, in corpus cavernosal smooth muscle cells. *BJU International*.

[42] Muzaffar S., Shukla N., Srivastava A., Angelini G. D., Jeremy J. Y. (2005). Sildenafil citrate and sildenafil nitrate (NCX 911) are potent inhibitors of superoxide formation and gp91^phox^ expression in porcine pulmonary artery endothelial cells. *British Journal of Pharmacology*.

[43] Chan S. H. H., Wu C.-A., Wu K. L. H., Ho Y.-H., Chang A. Y. W., Chan J. Y. H. (2009). Transcriptional upregulation of mitochondrial uncoupling protein 2 protects against oxidative stress-associated neurogenic hypertension. *Circulation Research*.

[44] Touyz R. M., Chen X., Tabet F. (2002). Expression of a functionally active gp91phox-containing neutrophil-type NAD(P)H oxidase in smooth muscle cells from human resistance arteries: regulation by angiotensin II. *Circulation Research*.

[45] Muzaffar S., Jeremy J. Y., Sparatore A., del Soldato P., Angelini G. D., Shukla N. (2008). H 2S-donating sildenafil (ACS6) inhibits superoxide formation and gp91 phox expression in arterial endothelial cells: role of protein kinases A and G. *British Journal of Pharmacology*.

